# Spontaneous rupture of enormous renal angiomyolipoma in a pregnant tuberous sclerosis patient: a rare case

**DOI:** 10.1186/s12882-020-02124-w

**Published:** 2020-10-31

**Authors:** Zechuan Liu, Yinghua Zou, Tianshi Lv, Haitao Guan, Zeyang Fan

**Affiliations:** Department of Interventional and Vascular Surgery, Peking University First Hospital, Beijing, China

**Keywords:** Renal angiomyolipoma, Tuberous sclerosis, Pregnant, Rupture, Selective arterial embolization, Prenatal abdominal ultrasound, Aseptic liquefaction necrosis

## Abstract

**Background:**

Renal angiomyolipoma (RAML) is a rare benign kidney tumour comprised of adipose tissue, smooth muscle, and blood vessels. It can cause fatal complications if it ruptures. Although there have been reports of RAMLs rupturing, it is unusual to see RAMLs rupture during pregnancy, especially in pregnant women with tuberous sclerosis (TSC). Moreover, we reported a rare complication after selective arterial embolization (SAE) for the first time, which called aseptic liquefaction necrosis.

**Case presentation:**

The case is a 16-week-pregnant woman with TSC who presented with severe flank pain, which was secondary to the rupture of a large, previously unknown RAML. This was confirmed by emergency computed tomography and successfully treated with selective arterial embolization after the patient received counselling and provided prior informed written consent for medical termination of pregnancy (MTP). The patient underwent abortion 3 weeks after the SAE. The patient required drainage 2 months after the SAE because of aseptic liquefaction necrosis. During follow-up, the patient’s lesion remained stable.

**Conclusion:**

RAML rupture is a rare but rather serious complication in pregnant tuberous sclerosis patients. Selective arterial embolization (SAE) should be performed immediately, and the status of the pregnancy needs to be assessed by a multidisciplinary team. We also report for the first time the rare complication of aseptic liquefaction necrosis after SAE of RAML, for which percutaneous drainage is effective.

## Background

Renal angiomyolipoma (RAML) is a rare benign kidney tumour that originates from perivascular epithelioid cells, and it is even rarer for it to occur during pregnancy. Renal AML is a mesenchymal neoplasm made up of 3 components in varying proportions, including adipose tissue, smooth muscle, and blood vessels. Approximately 20% of AML patients have TSC, which is an autosomal dominant disease with an estimated prevalence of 1 in 20,000 [1, 2]. Most patients with RAML are asymptomatic and are only occasionally diagnosed, but some patients will have a fatal complication, such as rupture, bleeding and hypovolemic shock. For asymptomatic patients, a cut-off point of 4 cm, which was first reported in 1986 [3], is traditionally accepted to distinguish active surveillance and management. However, this standard is currently considered controversial, and several studies have noted that it might lead to overtreatment [4, 5]. In regard to Wunderlich syndrome, it manifests as acute nontraumatic flank or abdominal pain, palpable flank masses and ultimately fulminant hypovolemic shock [6].

## Case presentation

**Fig. 1:**
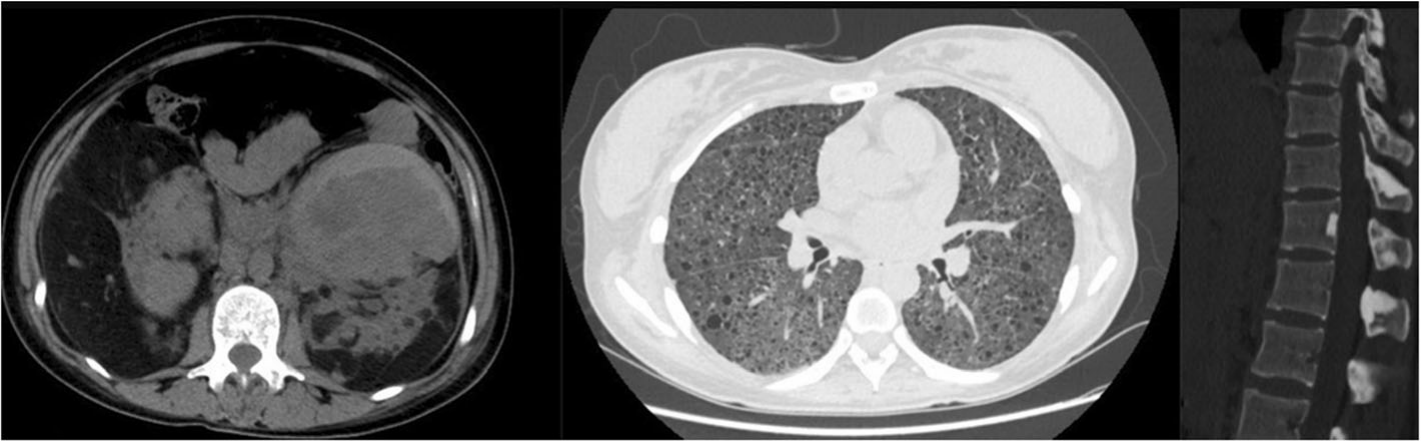
**a** Ruptured angiomyolipoma of the left kidney with subcapsular haematoma ahead of the mass; **b** diffuse ballonet in the lungs; **c** sclerotic bone lesions of the vertebra

In the 16th week of pregnancy, a 39-year-old patient (gravida 2 para 0) was admitted due to severe flank pain and wheezing. Physical examination showed palpable abdominal masses and multiple facial angiofibromas. Laboratory examination revealed RBC 3.13 × 10^12^/L, Hb 79 g/L, WBC 10.8 × 10^9^/L, creatinine 75.3 μmol/L, haematuria (+++) and type I respiratory failure with PaO2 56 mmHg and PaCO2 26 mmHg. Abdominal contrast-enhanced CT revealed bilateral huge RAML with the left one ruptured. The maximum diameters of the left and right kidney lesions were 21.7 cm and 21.2 cm, respectively. The diagnosis of AML was definite due to the fat composition. In addition, the HRCT showed many small, thin-walled cystic lesions and sclerotic bone lesions (Fig. 1). The patient had 2 pregnancies and 0 live births in the past. She miscarried in 2015 with no obvious cause and underwent uterine curettage in 2016 due to embryonic arrest. In addition, the patient had mild limitations of physical activity and she had epilepsy in childhood, but this symptom disappeared in adulthood.

**Fig. 2:**
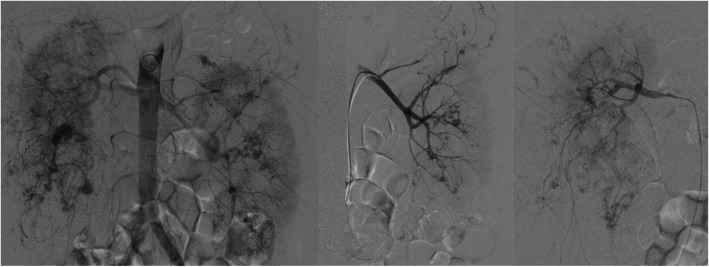
Angiography shows bilateral multiple aneurysms and tumour staining

Because of the unstable haemodynamics, bilateral renal SAE was performed emergently after relevant auxiliary examinations. Multiple lobular tumour staining and aneurysms could be observed during angiography (Fig. 2). Lipiodol and gelatine sponge were mixed to embolize the feeding arteries, and a coil was supplemented to embolize the branch artery of the left kidney. Following the procedure, the patient showed moderate pyrexia (38.2 °C) and mild flank pain. These symptoms were improved after supportive therapy. During the hospitalization, the patient completed the examinations of brain MRI and echocardiography, which showed cortical dysplasia and cardiac rhabdomyoma. It was 3 weeks later that medical termination of the pregnancy was completed because of the anaemia and hypoproteinaemia.

**Fig. 3:**
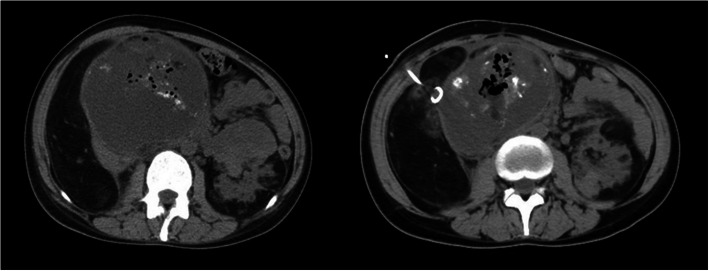
Liquefaction necrosis and percutaneous drainage in the right RAML 2 months after TACE

**Fig. 4:**
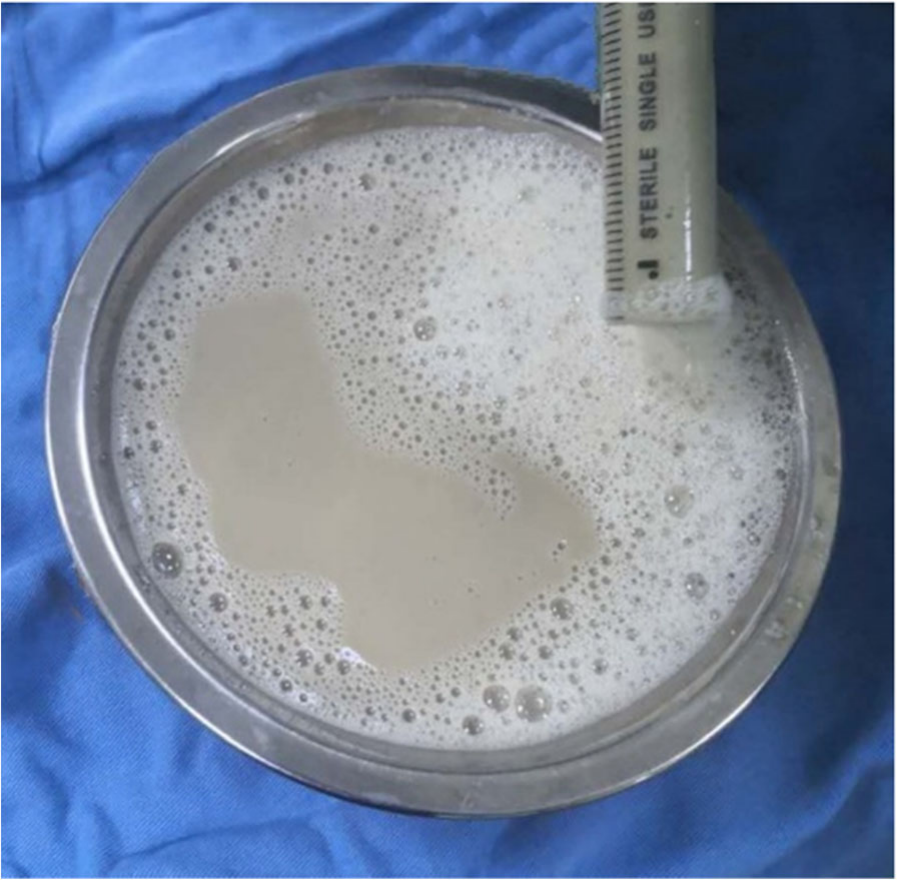
The fluid drained from the cyst; bacterial culture was negative

The patient was admitted a second time 2 months after SAE, presenting with abdominal pain and a high fever (39.4 °C) without shivering and expiratory dyspnoea. Laboratory examination showed WBC 13.9 × 10^9^/L and neutrophils 88.9%. The multiple blood gases were stable during the visit, with PaO2 54–60 mmHg and PaCO2 24–26 mmHg. The abdominal CT showed an “abscess” had formed in the right kidney (12.9 × 10.5 × 12.4 cm, Fig. 3). We performed ultrasound-guided puncture and drained a total of 700 ml of fluid (Fig. 4). The drainage fluid was grey with no odour, and the aerobic and anaerobe cultures were negative. The patient’s symptoms were improved after the operation, and her temperature dropped below 38 °C (WBC 7.6 × 10^9^/L).

## Discussion and conclusions

Rupture is the most serious complication of RAML and is also an important source of morbidity and mortality in TSC [7]. The features of patient in this case are as follows: facial angiofibroma (≥3), cortical dysplasia, cardiac rhabdomyoma (anterior wall of the left ventricle, 0.9 × 1.3 cm), lymphangioleiomyomatosis (LAM), renal angiomyolipoma, liver angiomyolipoma (suspected) and sclerotic bone lesions, which met the diagnostic criteria of TSC. During pregnancy, RAML tends to grow faster and to be vulnerable to rupture. Although a bleeding AML during pregnancy is a rare event, this condition has potentially devastating consequences, with significant maternal and foetal mortality. The mechanisms have lacked a fundamental theoretical basis so far. It is accepted that the main factor involved in the rupture of RAML is the elevated expression of oestrogen and progesterone receptors in tumours [8], and Boorjian et al. has reported that patients with TSC are significantly more likely to express both oestrogen receptor and progesterone receptors on RAMLs than in sporadic RAMLs [9]. In these settings, RAMLs with TSC tend to grow and rupture during pregnancy. In addition, increased maternal circulation and abdominal pressure in pregnancy also play an important role in the rupture of RAML [10].

It is clear that early detection and diagnosis of RAML may decrease the occurrence of serious complications. A routine abdominal ultrasound should be done before pregnancy, and evaluation for facial rash may facilitate early diagnosis [11]. In the present case, the patient did not accept abdominal ultrasound until she had abdominal pain after the retroperitoneal haematoma had formed. Although Wunderlich syndrome is not specific, abdominal ultrasound examination is necessary if the patient has recurrent abortion or Wunderlich syndrome. Of course, obstetric causes, such as ectopic pregnancy, placenta previa and placental abruption, should be differentiated first.

The management should be individualized based on the patient’s haemodynamic status, weeks of gestation and the association with TSC [12]. Patients with stable haemodynamics can be followed up from any trimester until term pregnancy [13], and periodic abdominal ultrasound should be implemented during surveillance. Patients with unstable haemodynamics and an expanding haematoma need to be operated on as an emergency. Surgery and selective arterial embolization are optional. Historically, there was a greater trend towards surgery over embolization for renal AMLs, but the circumstances have changed, especially in cases of acute haemorrhage [8]. SAE was selected in this case due to the acute rupture and unstable haemodynamics. mTOR inhibitors were not used in this patient because relevant evidence on pregnant safety is still lacking.

On the preoperative CT, the bilateral RAMLs had reached the level of the pubic symphysis, which could be pressed on by the enlarged uterus as the pregnancy progressed. Further, we found multiple aneurysms in the process of SAE, which also indicated a high risk of rupture. For the above reasons, the patient chose abortion after being informed of the possible risks. Continuous oxygen inhalation was given during her hospital stay. When she was discharged from hospital, we believed it was unnecessary to initiate long-term home oxygen therapy because the symptom of wheezing was partly due to the pregnancy. We undertook active surveillance for her blood oxygen level and HRCT.

Two months after TACE, we performed percutaneous drainage because liquefaction necrosis (negative culture) formed. Liquefaction necrosis after TACE is unusual in RAMLs, and its formation mechanism is not clear. According to our single-centre experience, it may be associated with tuberous sclerosis, embolism material (gelatine sponge), and solid embolism. Drainage is necessary for these patients, and we were able to apply antibiotics empirically because it is difficult to differentiate liquefaction necrosis from an abscess before bacterial culture. In the meantime, her symptom of wheezing was relieved in this visit.

Foetal gestation post-embolization is an issue we were concerned about. The risk of malformation is high when a foetus is less than 10 weeks old, and the cancer risk to the foetus increases as a result of radiation exposure during SAE [14]. However, successful term delivery post-embolization is feasible even when it is applied in the first trimester [10, 13]. It needs to be managed in a multidisciplinary manner to assess if the gestation could continue. Of course, precautions should be taken to reduce radiation exposure to the foetus, such as radiation shielding, minimal angiography imaging and low-dose-rate fluoroscopy [15].

RAML rupture is a rare but rather serious complication in pregnant tuberous sclerosis patients. Selective arterial embolization (SAE) should be performed immediately, and the status of the pregnancy needs to be assessed by a multidisciplinary team. We also report for the first time the rare complication of aseptic liquefaction necrosis after SAE of RAML, for which percutaneous drainage is effective.

## Data Availability

All data collected from this patient were obtained from Peking University First Hospital and are available in this paper.

## Abbreviations

RAML: Renal angiomyolipoma
TSC: Tuberous sclerosis
SAE: Selective arterial embolization
MTP: Medical termination of pregnancy
